# Supplementary material for: Antiproliferative and Cytotoxic Cytochalasins from Sparticola triseptata Inhibit Actin Polymerization and Aggregation
Source: J Fungi (Basel). 2022 May 25;8(6):560. doi: 10.3390/jof8060560 (PMC9225350; doi:10.3390/jof8060560)
Supplement: Supplementary file 1 [file jof-08-00560-s001.zip › jof-1723539-supplementary.pdf]

SUPPLEMENTARY MATERIAL FOR

Antiproliferative and cytotoxic cytochalasins from *Sparticola triseptata* inhibit actin polymerization and aggregation

Katherine Yasmin M. Garcia,[1,2] Mark Tristan J. Quimque,[1,2,3] Christopher Lambert,[4,5] Katharina Schmidt,[5] Gian Primahana,[4,6] Theresia E. B. Stradal,[5] Andreas Ratzenböck,[7] Hans-Martin Dahse,[8] Chayanard Phukhamsakda,[9,10] Marc Stadler,[4,11] Frank Surup,[4,11*] and Allan Patrick G. Macabeo*[,2]

[1]The Graduate School, University of Santo Tomas, España Blvd., 1015 Manila, Philippines;

[2]Laboratory for Organic Reactivity, Discovery and Synthesis (LORDS), Research Center for the Natural and Applied Sciences, University of Santo Tomas, España Blvd., 1015 Manila, Philippines;

[3]Chemistry Department, College of Science and Mathematics, Mindanao State University–Iligan Institute of Technology, Tibanga 9200, Iligan City, Philippines

[4]Department of Microbial Drugs, Helmholtz Centre for Infection Research and German Centre for Infection Research (DZIF), partner site Hannover/Braunschweig, Inhoffenstrasse 7, 38124 Braunschweig, Germany;

[5]Department of Cell Biology, Helmholtz Centre for Infection Research (HZI), Inhoffenstraße 7, 38124 Braun-schweig, Germany

[6]Research Center for Chemistry, National Research and Innovation Agency (BRIN), Kawasan Puspitek, Serpong, Tangerang Selatan 15314, Indonesia

[7]Institut für Organische Chemie, Universität Regensburg, Universitätstrasse 31, D-93053 Regensburg, Germany

[8]Leibniz-Institute for Natural Product Research and Infection Biology, Hans-Knöll-Institute (HKI), D-07745 Jena, Germany

[9]Center of Excellence in Fungal Research, Mae Fah Luang University, Chiang Rai 57100, Thailand

[10]Institute of Plant Protection, College of Agriculture, Engineering Research Center of Chinese Ministry of Education for Edible and Medicinal Fungi, Jilin Agricultural University, Changchun City, Jilin Province, People’s Republic of China, 130118

[11]Institute of Microbiology, Technische Universität Braunschweig, Spielmannstraße 7, 38106 Braunschweig, Germany

* Correspondence: frank.surup@helmholtz-hzi.de, agmacabeo@ust.edu.ph

LIST OF SUPPORTING INFORMATION

Page

Figure S1. ^{1}H NMR spectrum (MeOH-d_4, 600 MHz) of triseptatin (**1**) 4

Figure **S2.** ^{13}C NMR spectrum (MeOH-d_4, 600 MHz) of triseptatin (**1**) 4

Figure S3. HSQC-DEPT spectrum of triseptatin (**1**) 5

Figure **S4.** COSY spectrum of triseptatin (**1**) 5

Figure **S5.** HMBC spectrum of triseptatin (**1**) 6

Figure **S6.** ROESY spectrum of triseptatin (**1**) 6

Figure S7. HR-ESIMS spectrum of triseptatin (**1**) 7

Figure S8. Low energy conformers (> 1%) of (*S*)-**1** optimized at B3LYP/6-31G(d) (PCM/MeOH). 7

Figure S9. ^{1}H NMR spectrum (MeOH-d_4, 600 MHz) of deoxaphomin B (**2**) 8

Figure S10. ^{13}C NMR spectrum (MeOH-d_4, 600 MHz) of deoxaphomin B 8

(**2**) 9

9

Figure S11. ^{1}H NMR spectrum (MeOH-d_4, 600 MHz) of cytochalasin B (**3**)

10

Figure S12. ^{13}C NMR spectrum (MeOH-d_4, 600 MHz) of cytochalasin B

(**3**)

10

Figure S13. ^{1}H NMR spectrum (MeOH-d_4, 600 MHz) of *cis*-4-hydroxy-6-deoxyscytalone (**4**)

11

Figure S14. ^{13}C NMR spectrum (MeOH-d_4, 600 MHz) of *cis*-4-hydroxy-6-deoxyscytalone (**4**) 11

Figure S15. ^{1}H NMR spectrum (MeOH-d_4, 500 MHz) of 6-hydroxymellein

(**5**)

Figure S16. ^{13}C NMR spectrum (MeOH-d_4, 125 MHz) of 6-

hydroxymellein (**5**)

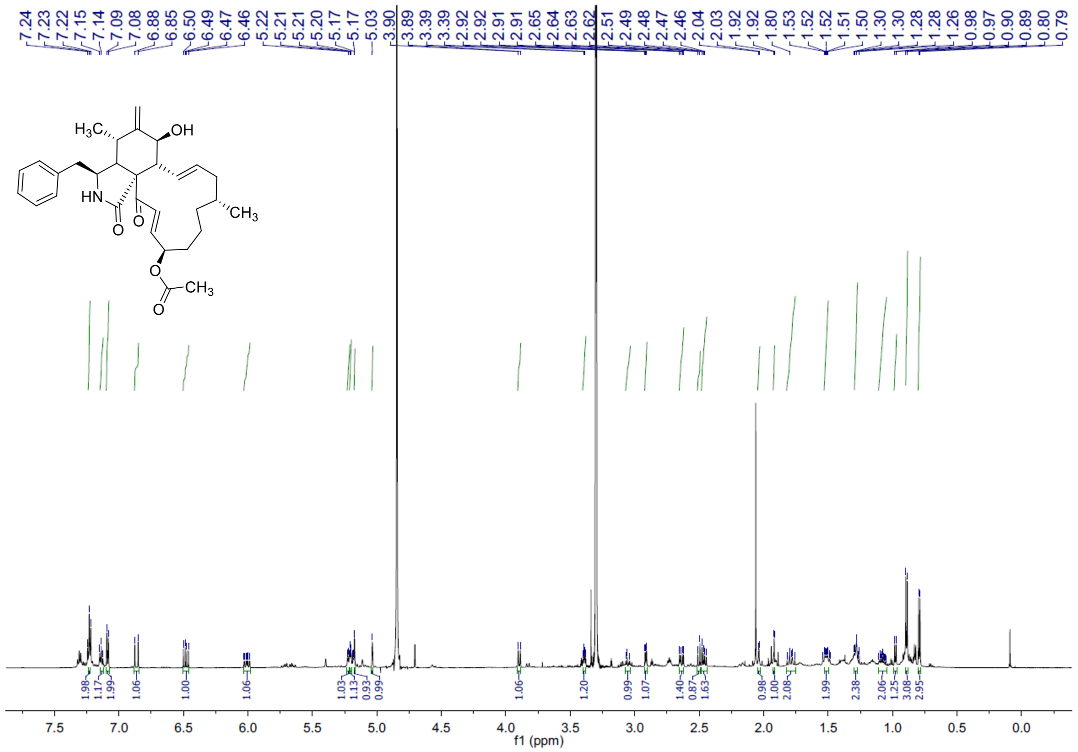

Figure S1. ^{1}H NMR spectrum (MeOH-d_4, 600 MHz) of triseptatin (**1**).

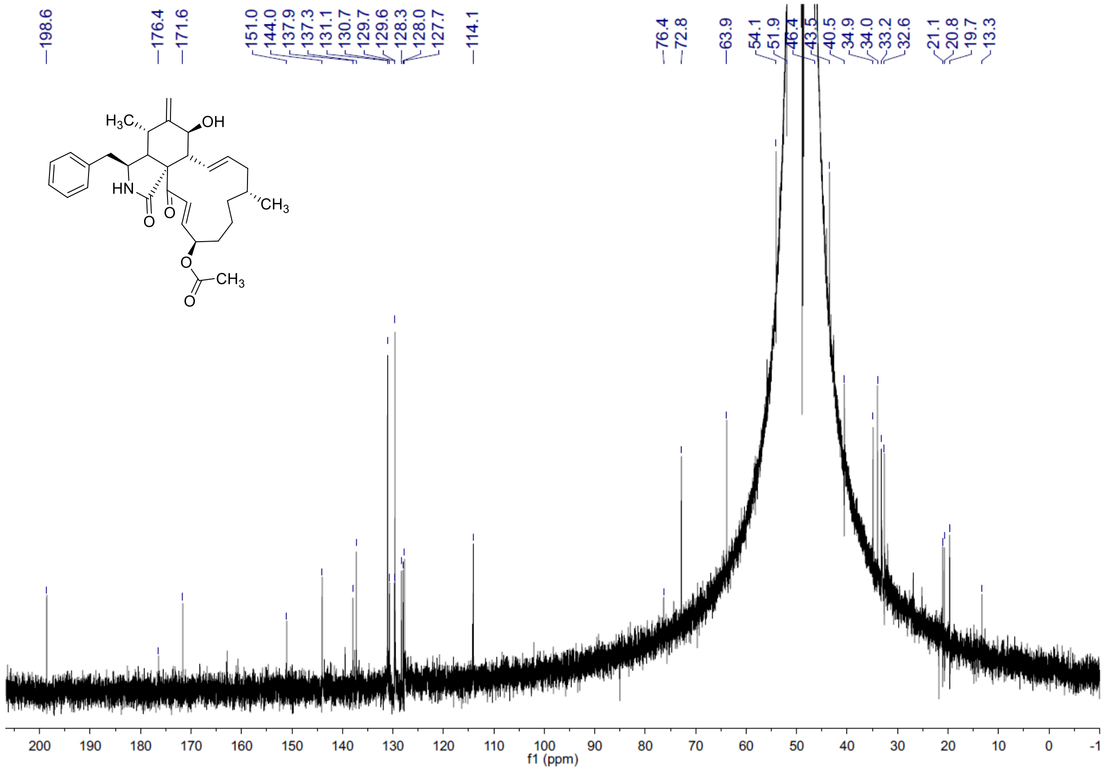

Figure S2. ^{13}C NMR spectrum (MeOH-d_4, 600 MHz) of triseptatin (**1**).

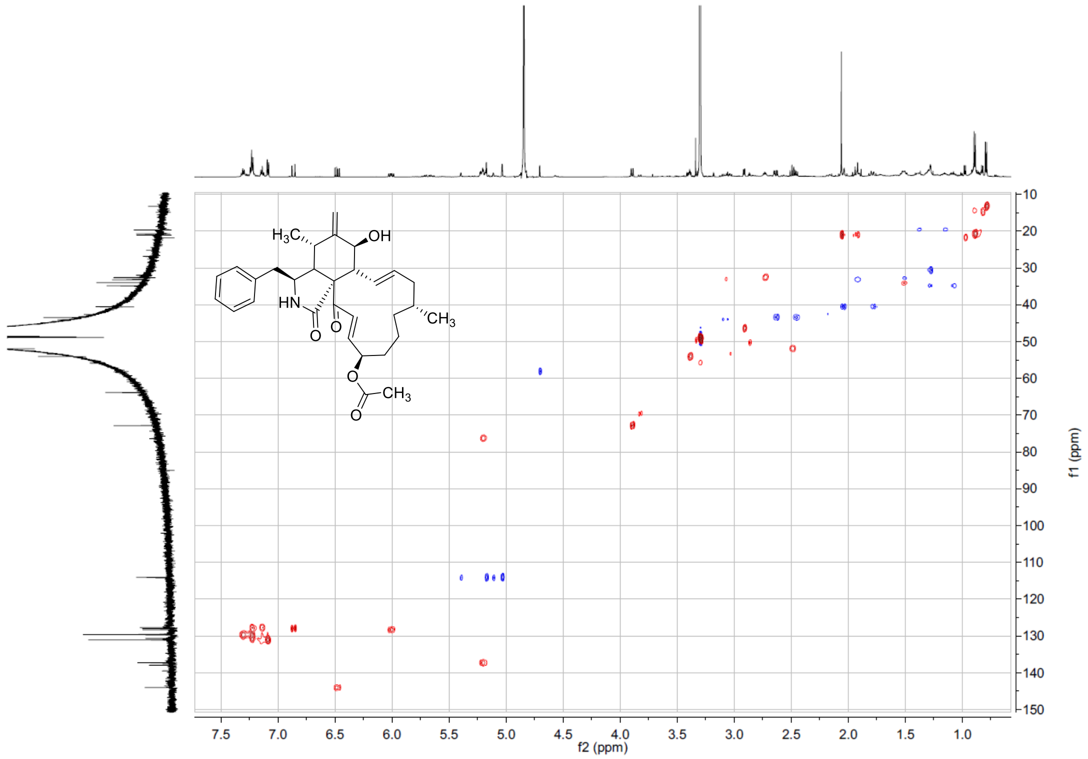

Figure S3. HSQC-DEPT spectrum of triseptatin (**1**).

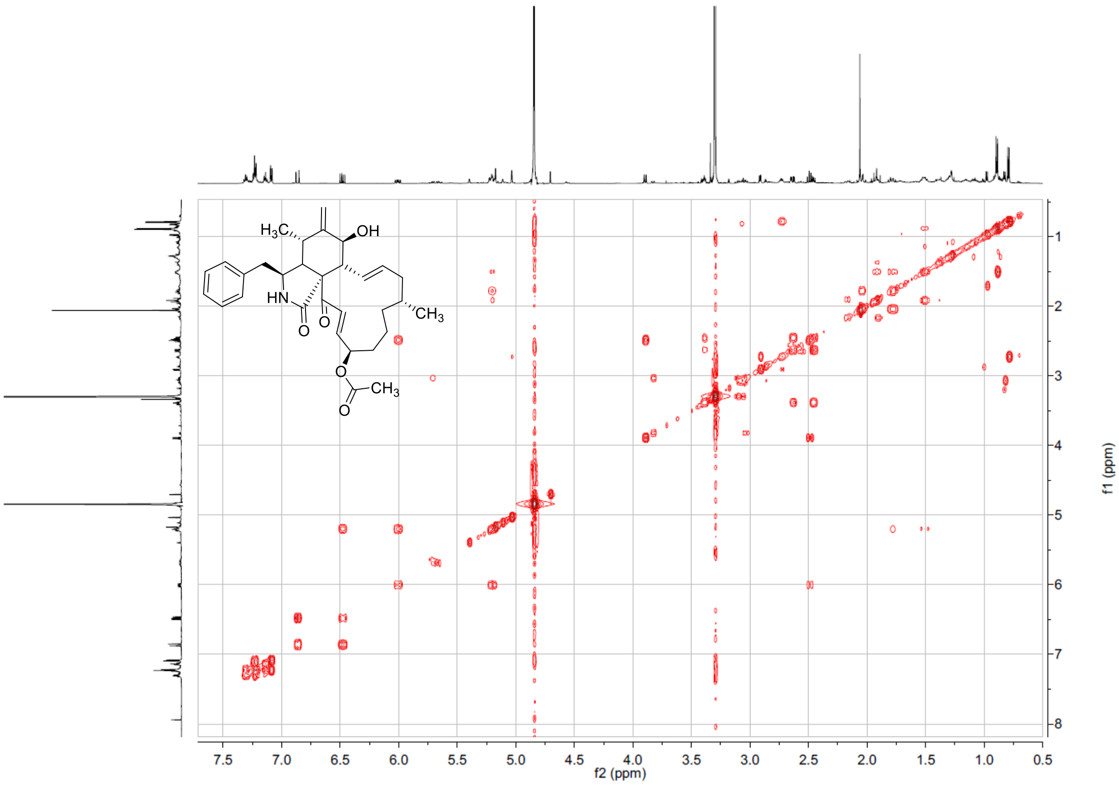

Figure S4. COSY spectrum of triseptatin (**1**).

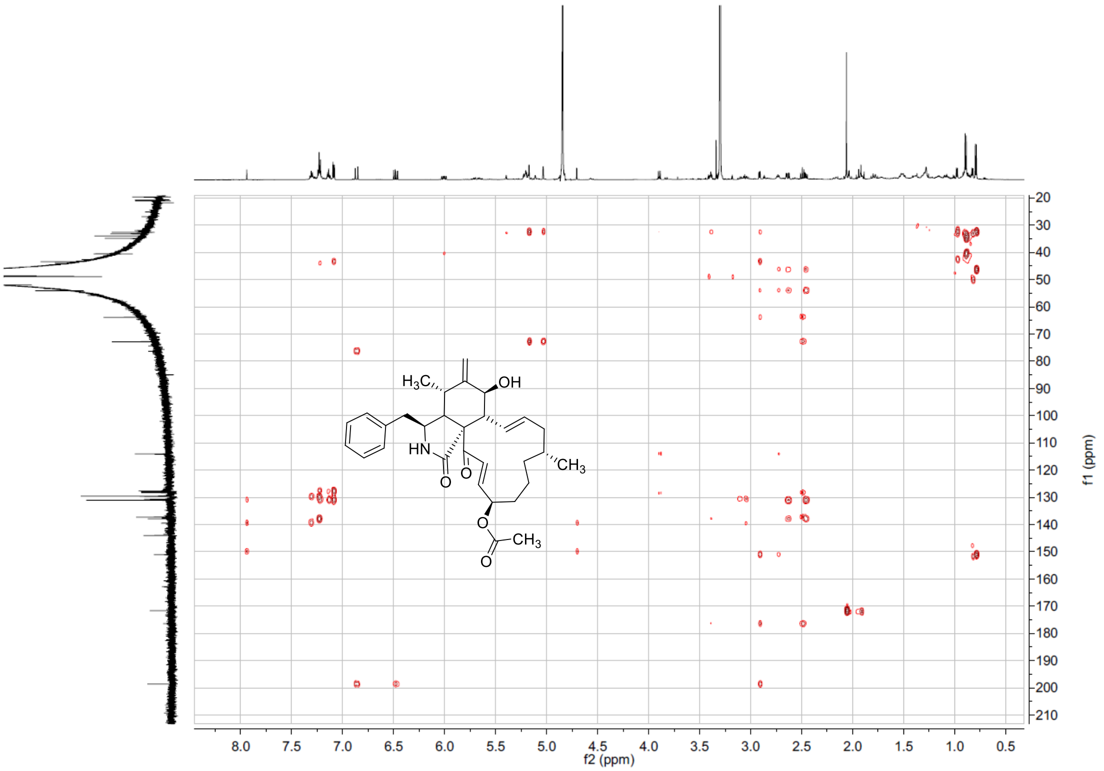

Figure S5. HMBC spectrum of triseptatin (**1**).

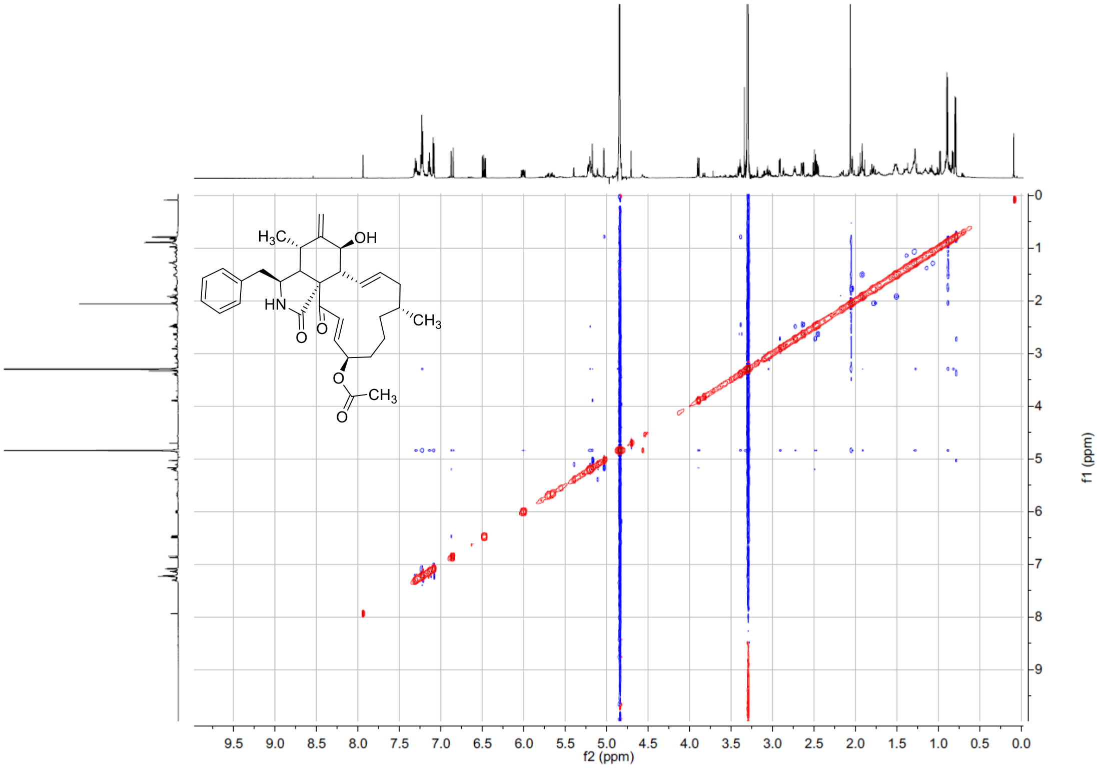

Figure S6. NOESY spectrum of triseptatin (**1**).

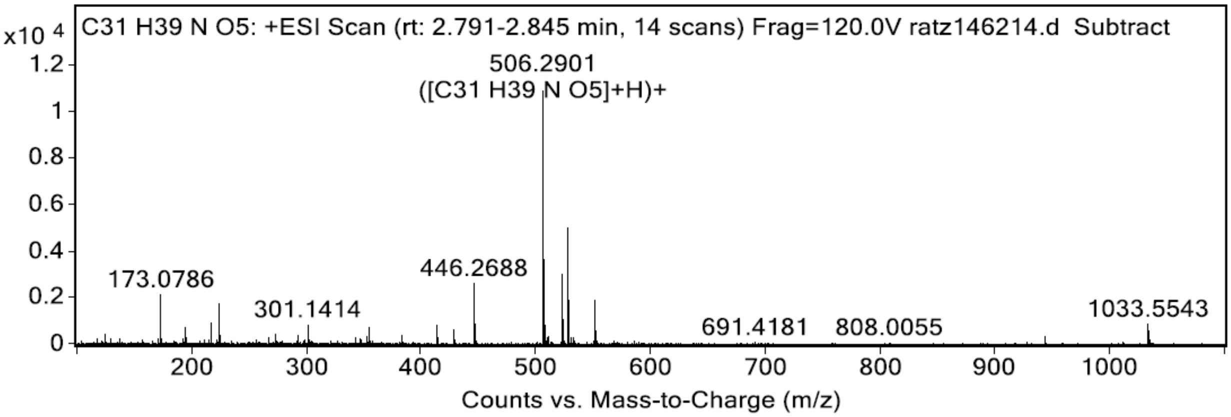

Figure S7. HR-ESIMS spectrum of triseptatin (**1**).

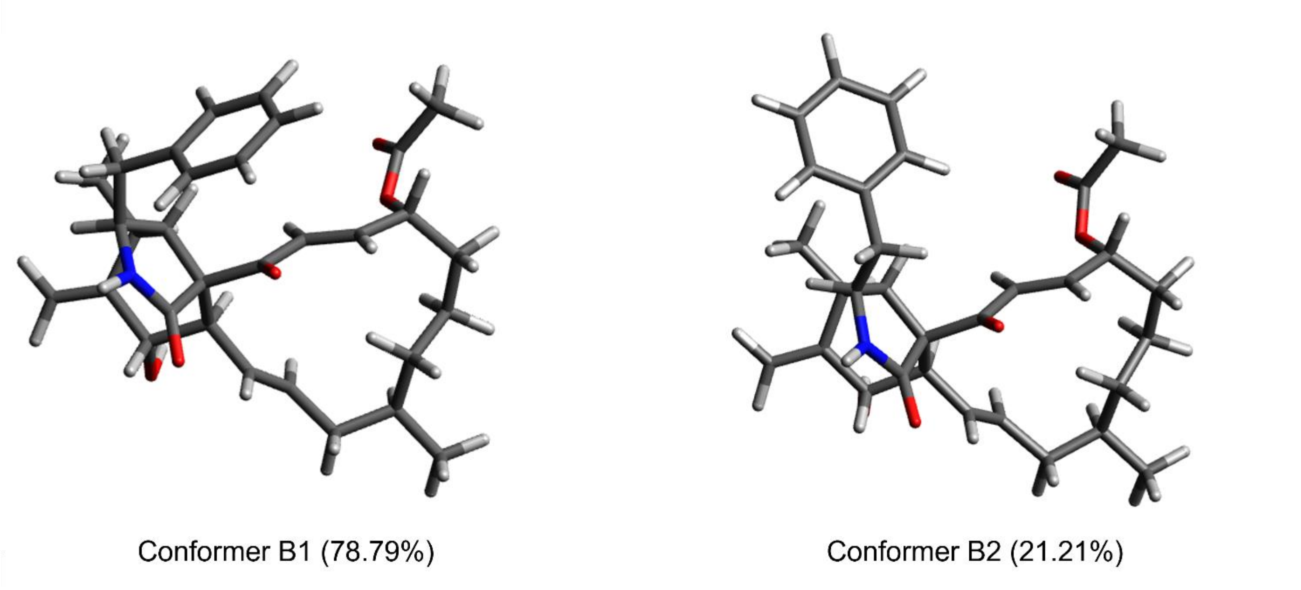

Figure S8. Low energy conformers (> 1%) of (*S*)-**1** optimized at B3LYP/6-31G(d) (PCM/MeOH).

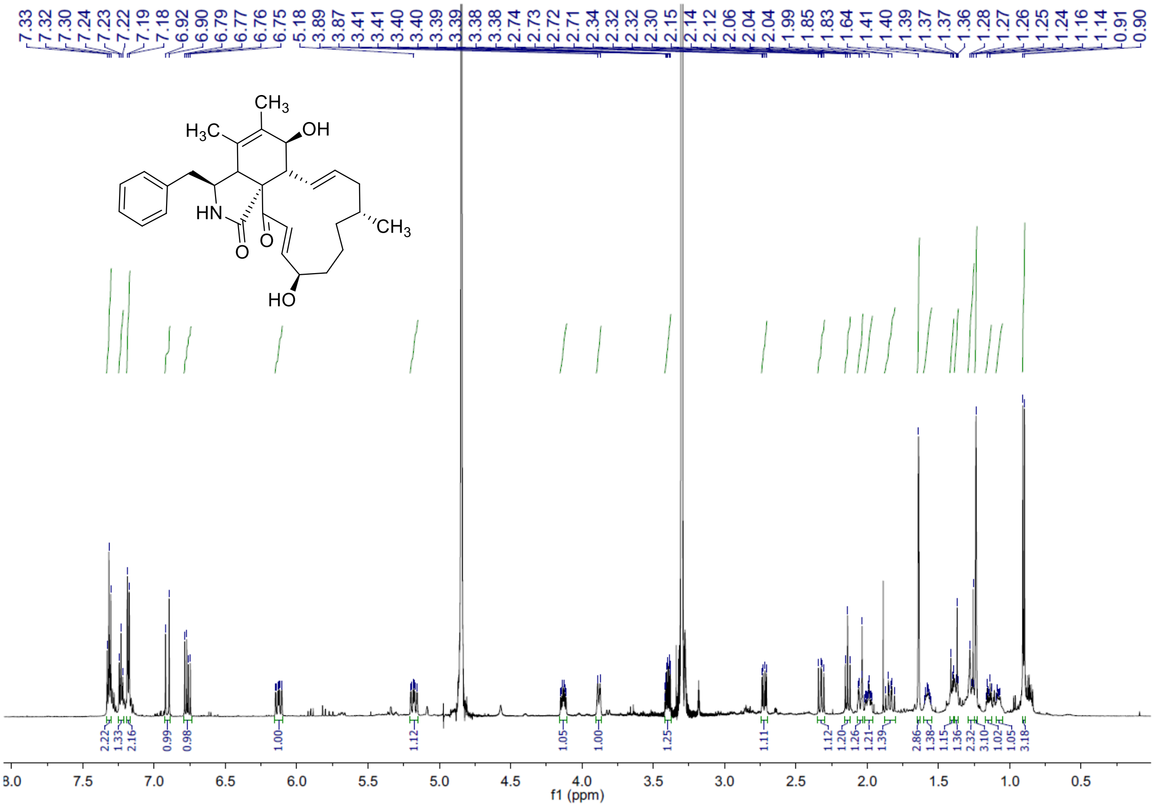

Figure S9. ^{1}H NMR spectrum (MeOH-d_4, 600 MHz) of deoxaphomin B (**2**).

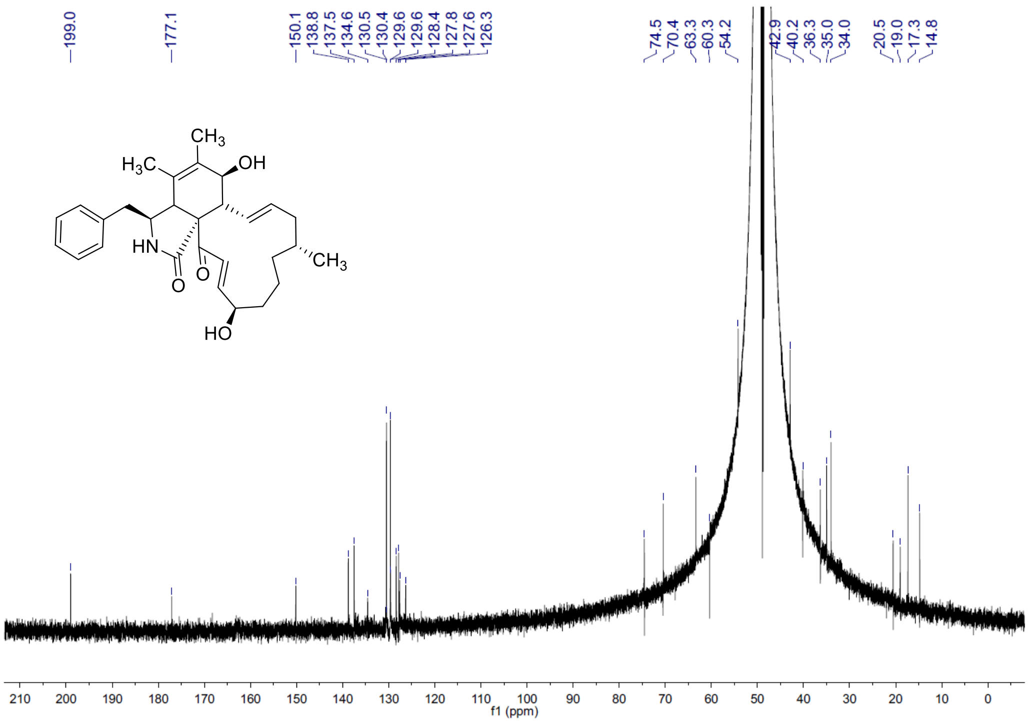

Figure S10. ^{13}C NMR spectrum (MeOH-d_4, 600 MHz) of deoxaphomin B (**2**).

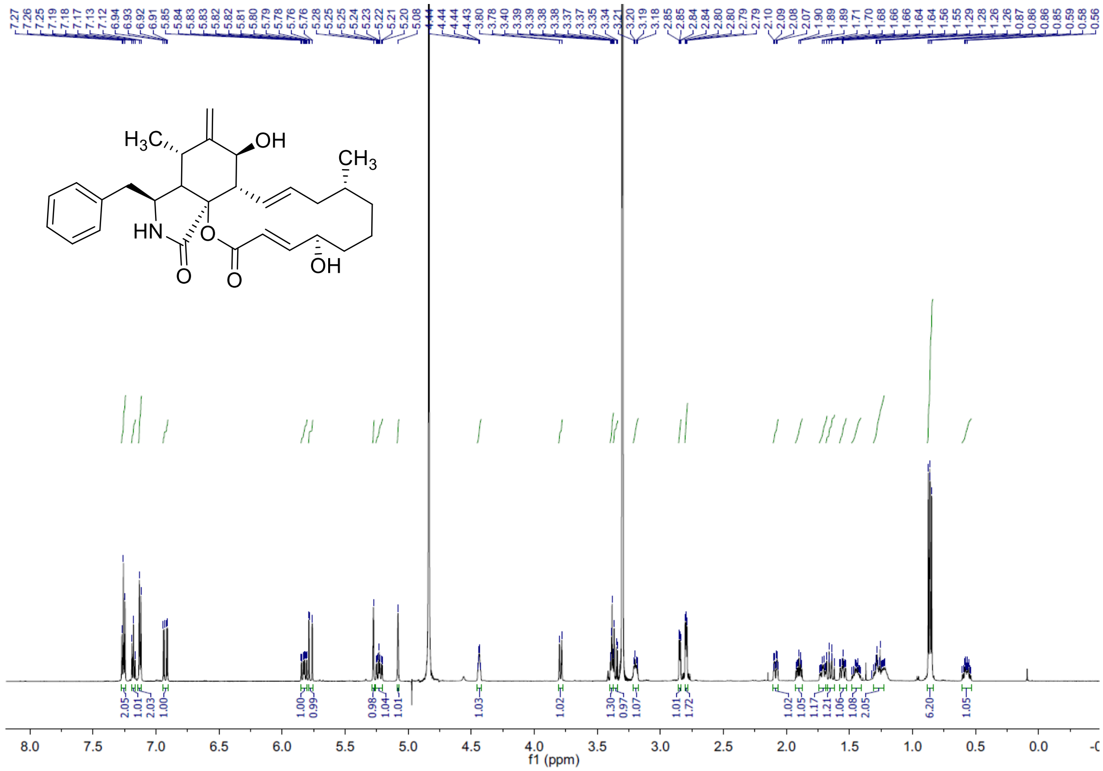

Figure S11. ^{1}H NMR spectrum (MeOH-d_4, 600 MHz) of cytochalasin B (**3**).

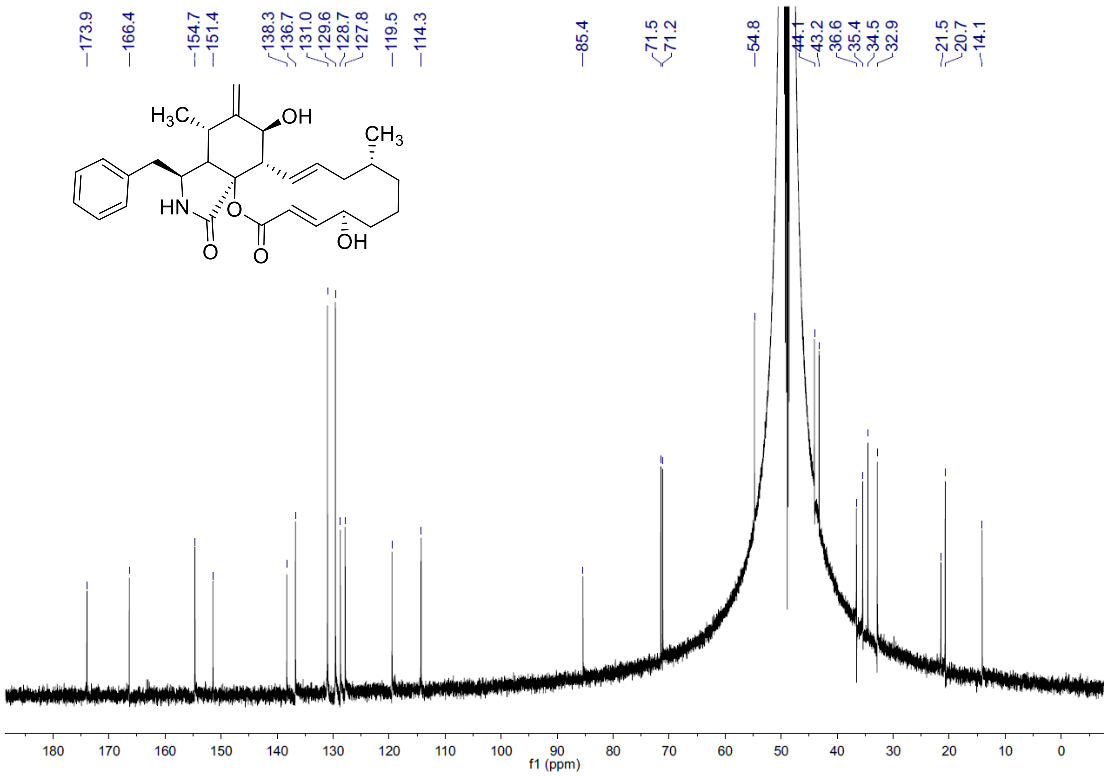

Figure S12. ^{13}C NMR spectrum (MeOH-d_4, 600 MHz) of cytochalasin B (**3**).

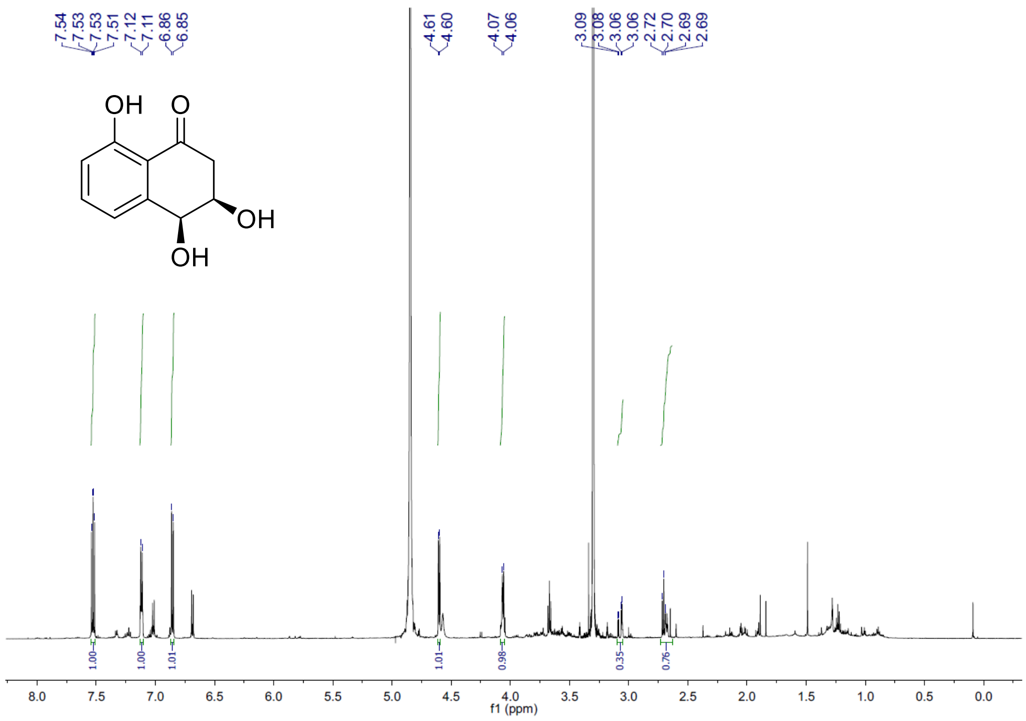

Figure S13. ^{1}H NMR spectrum (MeOH-d_4, 600 MHz) of *cis*-4-hydroxy-6-deoxyscytalone (**4**).

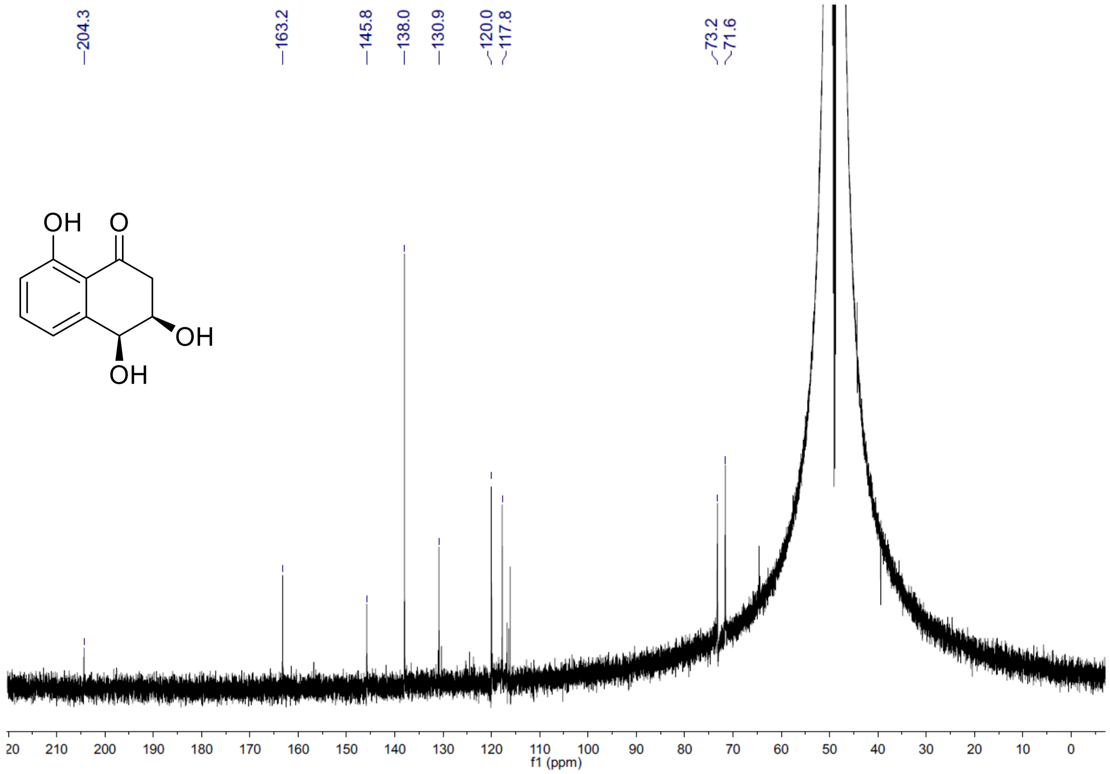

Figure S14. ^{13}C NMR spectrum (MeOH-d_4, 600 MHz) of *cis*-4-hydroxy-6-deoxyscytalone (**4**).

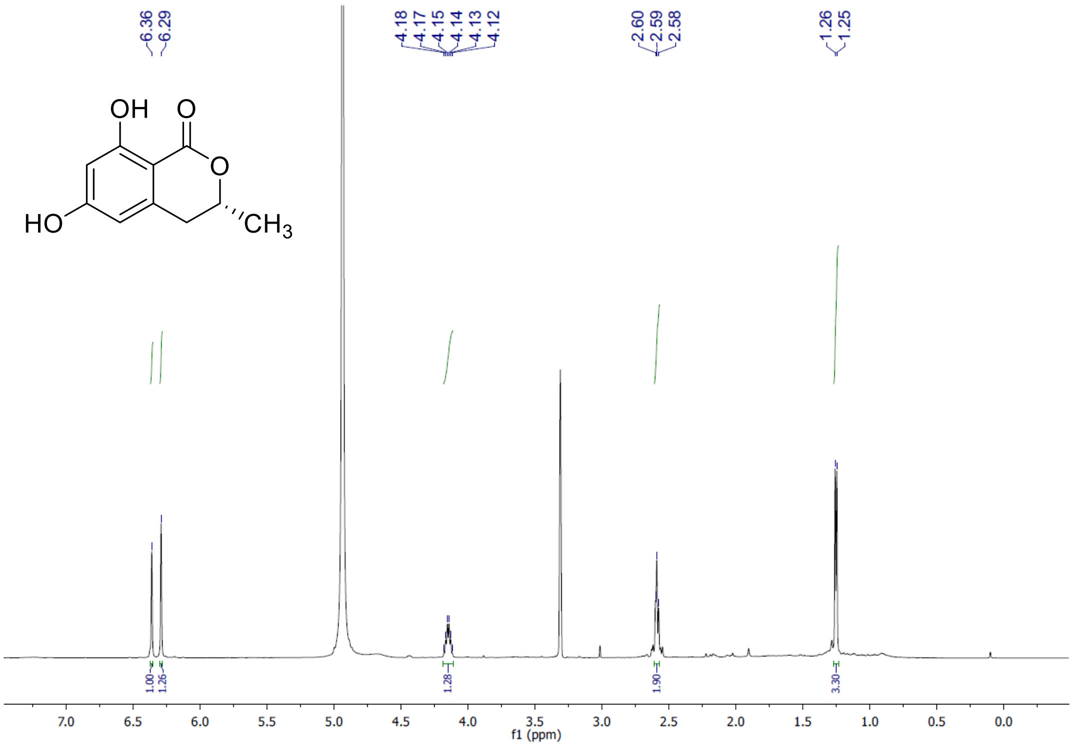

Figure S15. ^{1}H NMR spectrum (MeOH-d_4, 500 MHz) of 6-hydroxymellein (**5**).

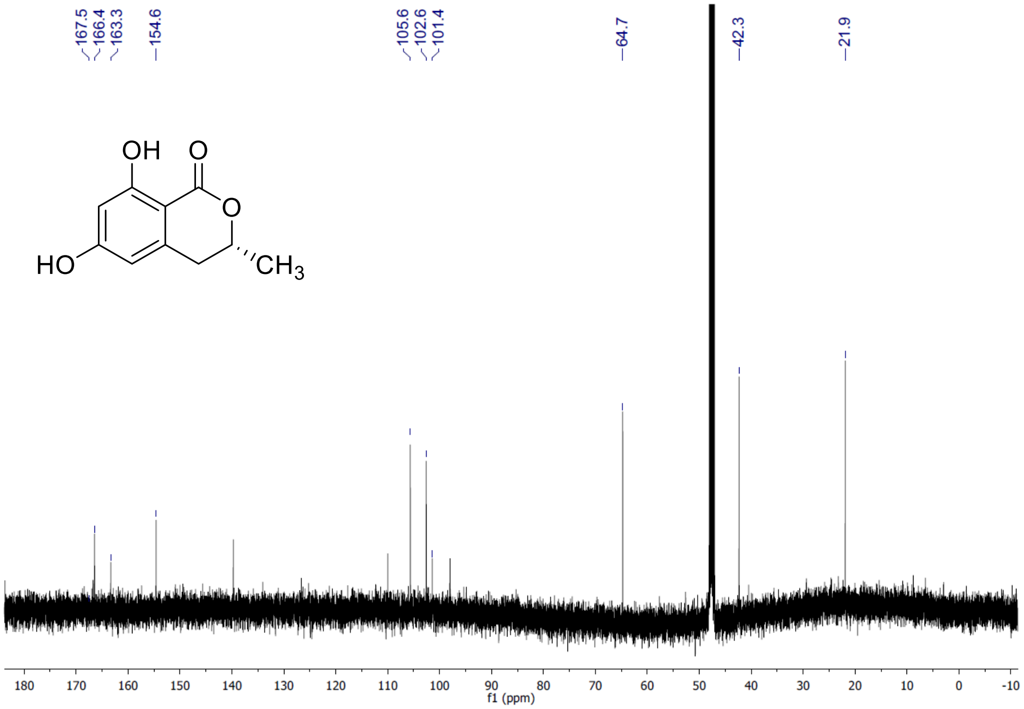

Figure S16. ^{13}C NMR spectrum (MeOH-d_4, 125 MHz) of 6-hydroxymellein (**5**).
